# Computed Tomographic Morphometry of the Internal Anatomy of Mandibular Second Primary Molars

**DOI:** 10.5005/jp-journals-10005-1313

**Published:** 2015-09-11

**Authors:** Ameet J Kurthukoti, Pranjal Sharma, Dinesh Francis Swamy, R Shashidara, Elaine Barretto Swamy

**Affiliations:** Professor and Head, Department of Pedodontics and Preventive Dentistry, Jodhpur Dental College and General Hospital, Jodhpur, Rajasthan, India; Ex-Postgraduate Student, Department of Pedodontics and Preventive Dentistry, Coorg Institute of Dental Sciences, Virajpet, Karnataka, India; Senior Lecturer, Department of Pedodontics and Preventive Dentistry, Vydehi Institute of Dental Sciences, Bengaluru, Karnataka, India; Professor and Head, Department of Oral Pathology, Coorg Institute of Dental Sciences, Virajpet, Karnataka, India; Lecturer, Department of Pedodontics and Preventive Dentistry, Goa Dental College and Hospital, Bambolim, Goa, India

**Keywords:** Area, Cross-sectional shape, Deciduous/anatomy and histology, Dental pulp cavity/anatomy and histology, Number of canals, Root canal therapy/instrumentation, Root dentin thickness, Spiral computed/methods, Tomography, Tooth.

## Abstract

**Need for the study:** The most important procedure for a successful endodontic treatment is the cleaning and shaping of the canal system. Understanding the internal anatomy of teeth provides valuable information to the clinician that would help him achieve higher clinical success during endodontic therapy.

**Aims:** To evaluate by computed tomography—the internal anatomy of mandibular second primary molars with respect to the number of canals, cross-sectional shape of canals, cross-sectional area of canals and the root dentin thickness.

**Materials and methods:** A total of 31 mandibular second primary molars were subjected to computed-tomographic evaluation in the transverse plane, after mounting them in a prefabricated template. The images, thus, obtained were analyzed using De-winter Bio-wizard® software.

**Results:** All the samples demonstrated two canals in the mesial root, while majority of the samples (65.48%) demonstrated two canals in the distal root. The cross-sectional images of the mesial canals demonstrated a round shape, while the distal canals demonstrated an irregular shape. The root dentin thickness was highly reduced on the distal aspect of mesial and mesial aspect of distal canals.

**Conclusion:** The mandibular second primary molars demonstrated wide variation and complexities in their internal anatomy. A thorough understanding of the complexity of the root canal system is essential for understanding the principles and problems of shaping and cleaning, determining the apical limits and dimensions of canal preparations, and for performing successful endodontic procedures.

**How to cite this article:** Kurthukoti AJ, Sharma P, Swamy DF, Shashidara R, Swamy EB. Computed Tomographic Morphometry of the Internal Anatomy of Mandibular Second Primary Molars. Int J Clin Pediatr Dent 2015;8(3):202-207.

## INTRODUCTION

Primary teeth exhibit anatomical differences from permanent teeth in terms of size and external and internal morphology. In comparison with permanent teeth, the relatively thin layer of mineralized hard tissue between the external and internal surfaces leads to rapid involvement of the dental pulp by the advancing caries. Nonetheless, conservation of primary teeth is deemed advantageous for maintenance of arch length and harmonized temporal and facial development of permanent teeth. Pulpectomy of primary teeth is indicated when the dental pulp is irreversibly inflamed or non-vital.^1^

The main objective of root canal therapy is thorough shaping and cleaning of all pulp spaces and its complete obturation with an inert filling material. The presence of an untreated canal may be a reason for failure. A canal may be left untreated because the dentist fails to recognize its presence. It is extremely important that clinicians use all the armamentaria at their disposal to locate and treat the entire root canal system. It is humbling to be aware of the complexity of the spaces we are expected to access, shape, clean and fill. We can take comfort in knowing that even under the most difficult circumstances, our current methods of root canal therapy result in an exceptionally high rate of success.^2^

Primary teeth may often show bizarre internal geometry of the pulp cavity, with features not commonly observed in permanent teeth, such as variable shapes in cross-section, accessory canals in furcation area and horizontal anastomoses; rendering them difficult subjects for endodontic therapy. Even in contemporary dental practice, the prevailing notion that bizarre and tortuous root canals of primary teeth may not be adequately negotiated, cleaned, shaped or filled, has brought about needless sacrifice of carious primary teeth.^1^

The evolution of the dentistry has been paralleled by that of the systems to study the internal anatomy of teeth (from conventional radiology, to taking impressions of the root canals, making serial cuts or using blocks of resin), with different levels of complexity and of sample precision. Novel technologies, such as computed tomography have been directed to analyze the internal geometry of root canals; employing reconstruction and visualization methods that allow the canals to be observed three-dimensionally for treatment without destroying the specimen under study.^3^

There is paucity of currently available literature on the internal anatomic variations of mandibular second primary molar teeth using contemporary noninvasive imaging technology. Hence, this study was undertaken to:

- Evaluate the number and shape of canals in mandibu-lar second primary molars.
- Evaluate the root canal cross-sectional area at three pre-determined levels, and the root dentin thickness in mandibular second primary molars.

## MATERIALS AND METHODS

A total of 31 mandibular primary second molar teeth extracted for therapeutic reasons, were washed under running water and all the soft tissue was scraped from their crown and root surfaces using a periodontal scaler. The teeth were then autoclaved and stored in distilled water in air-tight containers, according to ISO guidelines.^4^

**Fig. 1:**
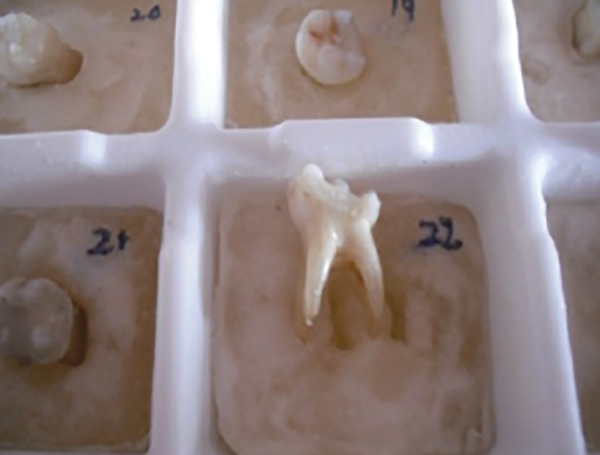
Sample embedded in acrylic jig permitting accurate repositioning

**Fig. 2:**
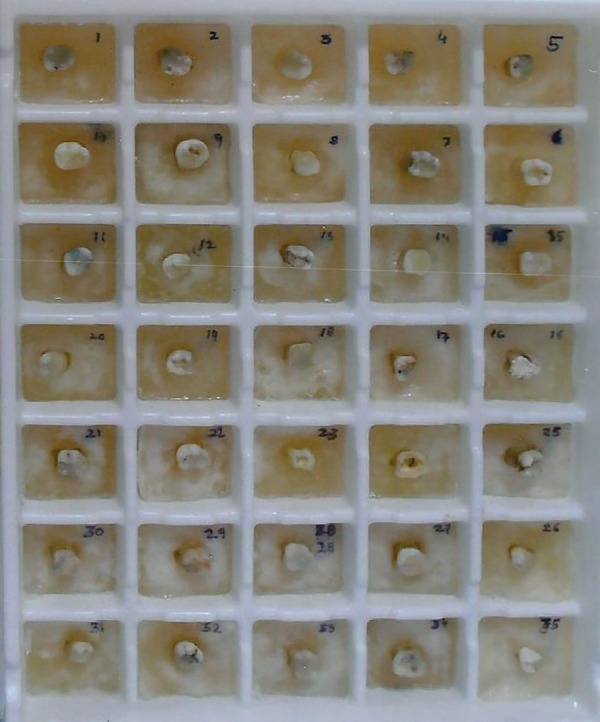
Prefabricated template bearing samples for scanning

**Fig. 3:**
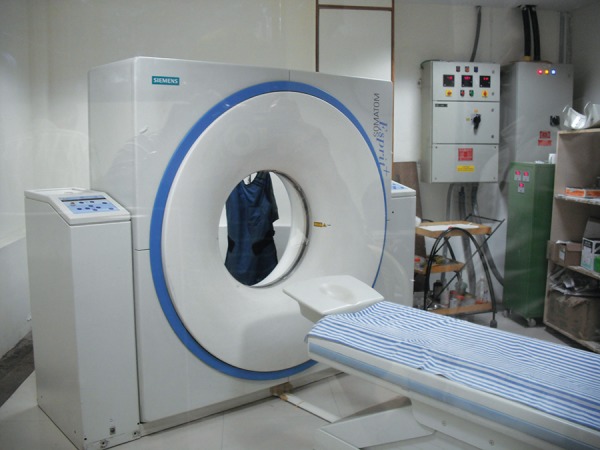
Spiral computed tomography unit (Siemens® SOMATOM Esprit + CT)

The root surfaces of the selected samples were coated with a thin layer of separating media and the teeth were embedded in the unset cold cure acrylic, such that each individual sample could be separated from their respective block and again repositioned, if needed (Fig. 1). The teeth were then aligned in a prefabricated template (Fig. 2), and a scan was performed in the transverse plane using a Siemens® SOMATOM Esprit + CT unit (Fig. 3).

**Fig. 4:**
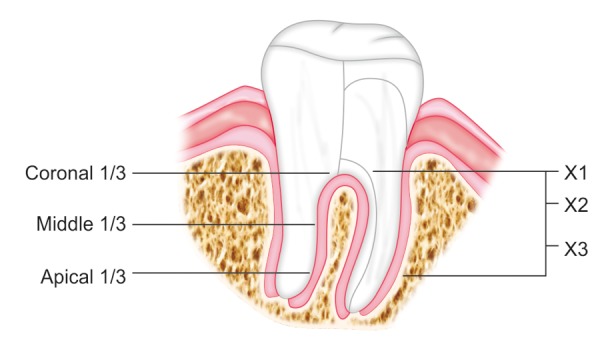
Levels at which root canal configuration were evaluated

The computed tomographic (CT) slices were then taken at three pre-determined levels. Initially, the length of each root of each of the specimen was determined using the CT slices beginning from the furcation till the root tip. This length was divided into three equal parts. The coronal two-thirds thus obtained was again divided into two subsequent equal parts. These three levels determined served as observational zones for evaluation of root canal configuration. Approaching from the furcation, the designated levels were (Fig. 4):

- The appearance of furcation was designated as X1.
- The mid-point of the coronal two-third root length measurement designated as X2.
- The apical most point of the coronal two-third root length measurement designated as level X3.

### Qualitative and Quantitative Parametric Assessment

**Fig. 5:**
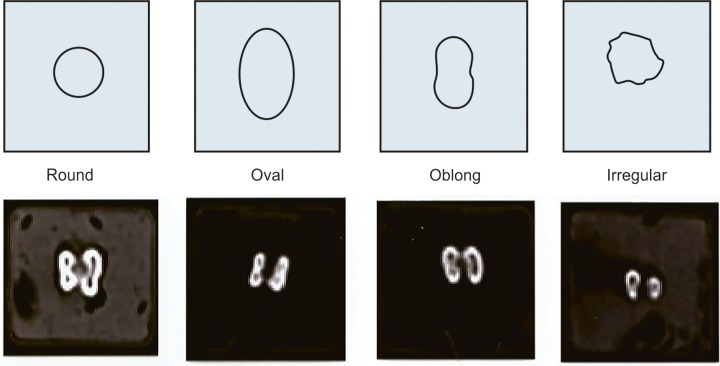
Image showing four cross-sectional shapes that RC morphology was categorized

Soft copies of the scanned images were obtained and analyzed using Dewinter Bio-wizard^®^ Software. The number of canals present in each root was counted. The root canal cross-sectional shapes at X1, X2 and X3 were recorded as (Fig. 5):

- Round
- Oval
- Oblong (i.e. an elongated oval shaped canal)
- Irregular with sharp angles or slot, including fins and perforations.

### Root Dentin Thickness

To assess the root dentin thickness, the shortest distance from the canal outline to the closest adjacent root surface was measured at each predetermined level using the imaging software.

To analyze interobserver agreement, 10% of the measurements were repeated at random and verified with an intraclass correlation coefficient.

## RESULTS

Table 1 shows the number of root canals observed in the mesial and distal roots of the mandibular second primary molars. While all the samples demonstrated two canals in the mesial root, 35.48% of the samples demonstrated a single canal in the distal root and 64.53% of the samples demonstrated two canals in the distal root.

### Cross-sectional Morphology of the Root Canals

Table 2 demonstrates that a round cross-sectional morphology was most commonly observed in the mesial canals at all three levels, i.e. 32.26% at X1, 61.29% at X2 and 87.1% at X3; while the other cross-sectional forms were observed in fewer specimens. However, in the distal canals, the round and irregular cross-sectional form was the predominantly observed cross-sectional form at level X1 (round: 38.71%, irregular: 35.48%). At level X2, irregular was the predominantly observed cross-sectional form (41.94%). Round was the predominant cross-sectional form at level X3 (61.29%).

**Table 1:** **Table 1:** Descriptive statistics showing the number of canals observed in the mesial and distal roots of the mandibular second primary molars

Mesial root		Single canal		0 (0%)	
		Two canals		31 (100%)	
Distal root		Single canals		11(35.48%)	
		Two canals		20(64.52%)	

**Table 2:** **Table 2:** Descriptive statistics showing the cross-sectional shape of canals observed in the mesial and distal roots at all the three predetermined levels of the mandibular second primary molars

		*Mesial root*								*Distal root*							
*Levels*		*Round*		*Oval*		*Oblong*		*Irregular*		*Round*		*Oval*		*Oblong*		*Irregular*	
X1		32.26%		32.26%		6.45%		29.03%		16.13%		38.71%		9.68%		35.48%	
X2		61.29%		0%		3.23%		35.48%		22.58%		16.13%		25.81%		35.48%	
X3		87.1%		0%		0%		12.9%		61.29%		6.45%		0%		32.26%	

### Mean Area of Root Canals

The mean area of the individual root canals at all the three levels was calculated in sq. mm. Table 3 demonstrates the cross-sectional area of canals observed in the mesial and distal roots at three pre-determined levels. While the mean area of the mesial canals at X1 was 3.61 sq. mm, at X2 it was 0.84 sq. mm and at X3 it was 0.43 sq. mm. However, the mean area of the distal canal at X1 was 4.40 sq. mm at X2 2.23 sq. mm and at X3 1.17 sq. mm.

### Remaining Root Dentin Thickness

Table 4 demonstrates the remaining root dentin thickness in the mesial and distal roots of the mandibular second primary molar at all the three pre-determined levels.

## DISCUSSION

Successful endodontic therapy stems from thorough canal debridement and effective filling of the root canal system, for which knowledge of morphology of the root canals is a critical prerequisite.^5^ Internal complexities of the root canal are genetically determined and have definitive importance in anthropology.^6^ These factors necessitate the identification of a method that accurately determines the root canal morphology.

There are numerous reports on the root canal morphologies of different populations, which is extremely important for an endodontist as well as general dental practitioners. Also of interest to us are the methods that have been used in these studies.^3^

**Table 3:** **Table 3:** Descriptive statistics showing the cross-sectional area (sq. mm) of canals observed in the mesial and distal roots at all the three predetermined levels of the mandibular second primary molars

		*Mesial root*								*Distal root*							
*Levels*		*Mean*		*SD*		*Max.*		*Min.*		*Mean*		*SD*		*Max.*		*Min.*	
X1		3.612		1.846		6.467		0.61		4.401		1.947		9.681		1.134	
X2		0.838		0.376		1.586		0.33		2.227		1.464		6.31		0.336	
X3		0.426		0.172		0.897		0.142		1.116		0.928		4.07		0.012	

**Table 4:** **Table 4:** Descriptive statistics showing the remaining root-dentin thickness of canals toward and away from the furcation observed in the mesial and distal roots at all the three predetermined levels of the mandibular second primary molars

				*Mesial root*								*Distal root*							
*Levels*				*Mean*		*SD*		*Max.*		*Min.*		*Mean*		*SD*		*Max.*		*Min.*	
X1		Away Furca		1.859		0.325		2.433		1.037		1.923		0.356		2.582		1.042	
		Towards Furca		1.112		0.360		2.117		0.569		1.118		0.343		1.767		0.572	
X2		Away Furca		1.439		0.228		1.983		1.027		1.510		0.319		1.889		0.423	
		Towards Furca		1.196		0.289		1.736		0.578		1.291		0.388		1.939		0.215	
X3		Away Furca		1.059		0.276		1.667		0.412		1.064		0.308		1.854		0.547	
		Towards Furca		0.804		0.252		1.238		0.31		0.851		0.339		1.51		0.265	

The methods most commonly used in analyzing the root canal morphology are canal staining and tooth clearing, conventional radiographs, digital and contrast medium-enhanced radiographic techniques, radiographic assessment enhanced with contrast media, and more recently computed tomographic techniques.^3^

The application of CT scans in endodontics was first reported by Tachibana and Matsumoto in 1990.^7^ A CT scan uses a fan-shaped beam and multiple exposures around an object to reveal the internal architecture of this object, thereby helping the clinician to view morphologic features as well as pathology from different three-dimensional (3D) perspectives. The distinct advantage of a CT scan is that it allows for 3D reconstruction of root canal systems. Computed tomography scanning has been suggested as the preferential imaging modality in difficult situations demanding localization and description of root canal systems because of its ability to render 3D information.^8,9^

In our study, the number of root canals visualized in the mesial roots of mandibular second primary molars was two, i.e. mesiobuccal and mesiolingual. However, wide variations were observed when it came to the evaluation of the number of canals in the distal root. While 35.48% of the distal roots presented with a single canal, 64.52% of the roots presented two canals, i.e. distobuccal and distolingual.

The incidence of two canals in the distal root of man-dibular second primary molars is determined by genetics and anthropometry. Previous researchers have reported 21 to 28% incidence of two canals in the distal roots of mandibular second primary molars.^10^ However, a more recent study reports incidence rates as high as 53%.^11^ Aminabadi et al have reported 100% incidence of two canals in Iranian population.^1^ There are several explanations for this disagreement, one of which may be ethnicity of the subjects and another may be the difference in techniques used for assessment of internal morphology.^1^

Together with diagnosis and treatment planning, knowledge of the canal morphology and its frequent variations is a basic requirement for endodontic success. Stressing the significance of canal anatomy, Peters et al reported that variations in canal geometry before shaping and cleaning procedures had more influence on the changes that occur during preparation than the instrumentation techniques themselves.^12^

The predominantly observed cross-sectional morphology in the mesial canals at all three pre-determined levels was round. However, the cross-sectional canal morphology in the distal canals was more irregular in the coronal and middle third, and predominantly round in the apical third. This highlights the need for greater emphasis on a standardized funnel shaped preparation along the entire length of the canal as there is wide variation in the cross-sectional morphologies at all three levels.

The second most common cross-sectional shape observed was the irregular shape, the least observed shapes being oblong and oval. These observations highlight the need for a biomechanical preparation procedure that results in a standardized shape to ensure satisfactory fill. Previous studies have found oval or triangular shape to be the predominant cross-sectional shape in mandibular primary molars.^11^

The cross-sectional area of the distal canals was greater than the mesial canals at all the three pre-deter-mined levels. This could be attributed to the ribbon/ irregular shape of distal canals. The root canal area assessment revealed that the canals showed a decrease in cross-sectional area, in accordance with the root canal external morphology and it’s taper from the coronal to apical third. Similar observations were made previously by other researchers as well.^11^

For successful endodontic therapy, satisfactory preparation of the coronal and middle third portion of the root canal is mandatory; as this allows the removal of interferences permitting better access to the apical third of the root canal. However, root perforation is a possible consequence of flaring that results in treatment failure.

Also, flaring the canal excessively to allow instrumentation with larger files decreases the root dentin thickness, thus increasing the possibility of vertical root fracture. Recent research permits the use of rotary endodontic file systems in primary teeth as well.^13^ Thus, the root dentin thickness (RDT) is important because the amount of root dentin remaining enables the endodontically treated teeth to resist fracture; so, more the removal of dentin during instrumentation, more is the potential for perforation or fracture.

The mesial canals of mandibular permanent first molars are not located in the center of the root and the areas between the canals, and also, between the canals and the furcation area have thin dentin walls; and are therefore called ‘danger zones’. Danger zones have less dentin in the ramification areas in comparison with the peripheral safe root areas. Therefore, over-preparing the cervical and middle thirds of the root canal might result in thinning of the dentinal walls and sometimes cause strip perforations in the furcation area.^14^ In addition, thin dentinal walls increase permeability and fracture rate of teeth.

Observations of the remaining dentin thickness point to the fact that RDT reduces as we move coronoapically and also that this thickness is minimal on the root side toward furcation as compared to the side away. Reports suggest that the thickness of the distal wall of mesial roots in the permanent mandibular molars was merely 1.2 to 1.3 mm, just 1.5 mm apical to the furcation.^15^ Currently, available literature limits the concept of danger zone to the mandibular first permanent molar teeth. However, our study emphasizes that the apical third of the root canals of deciduous mandibular second primary molars are highly fragile as well. The greater curvature of the roots and the reduced root dentin thickness toward the furcation make primary teeth vulnerable to root perforations and resultant complications. Hence, it would be prudent to brand these areas as ‘danger zones’ of the mandibular second primary molars.

These observations highlight the need for exercising caution while preparing the apical portion of the root canals of these mandibular second primary molars. These findings also raise serious questions regarding the use of some of the aggressive rotary file systems in primary molars, especially in the apical thirds of the root canals.

## CONCLUSION

Within the limitations of the study, the following conclusions can be drawn:

- The mesial roots of the mandibular second primary molars commonly demonstrate two root canals, while the distal root of these teeth demonstrated two canals in 64.52% of the samples.
- The cross-sectional shapes of both mesial and distal canals of the mandibular second primary molars demonstrated wide variations at the pre-determined coronal, middle and apical levels. While the mesial canals predominantly demonstrated a round cross-sectional shape at all three levels, the distal canals demonstrated irregular or round cross-sectional shapes in the coronal and middle third, and round shape in the apical third of the teeth.
- The cross-sectional area of both mesial and distal canals demonstrated a gradual reduction from coronal third to the apical third. However, the cross-sectional area of the distal canals was considerably larger than the combined area of both the mesiobuccal and mesiolingual canals.
- The root dentin thickness on the distal wall of the mesial root and the mesial wall of the distal roots were reduced highlighting an existing ‘danger zone’, that is more prone for fractures and perforations during biomechanical preparation. This dentin thickness was minimal in the apical third of these walls in both the roots.

This study was a modest attempt to gain insight into the internal anatomy of mandibular second primary molar teeth that would help the clinicians achieve better success in endodontic practice. This study however, did not consider the inter-racial differences commonly seen in root morphology among different populations. We recommend future studies considering these criteria.
